# Alterations of thyroid microbiota across different thyroid microhabitats in patients with thyroid carcinoma

**DOI:** 10.1186/s12967-021-03167-9

**Published:** 2021-11-30

**Authors:** Daofeng Dai, Yan Yang, Yong Yang, Tianfeng Dang, Jiansheng Xiao, Weibin Wang, Lisong Teng, Juan Xu, Jing Ye, Hongqun Jiang

**Affiliations:** 1 Jiangxi Otorhinolaryngology Head and Neck Surgery Institute, Department of Otorhinolaryngology Head and Neck Surgery, The First Affiliated Hospital of Nanchang University, Nanchang, China; 2 Department of Surgical Oncology, The First Affiliated Hospital, School of Medicine, Zhejiang University, Hangzhou, China; 3 Department of Otolaryngology Head and Neck Surgery, Jiangxi Provincial People’s Hospital Affiliated to Nanchang University, Nanchang, China; 4 Department of General Surgery, The First Affiliated Hospital of Nanchang University, Nanchang, China; 5 Pathology Department, Taizhou Hospital of Zhejiang Province Affiliated to Wenzhou Medical University, Linhai, China

**Keywords:** Thyroid cancer, Microbiome, Lymph node metastasis, Biomarker, *Sphingomonas*

## Abstract

**Background:**

In recent years, the incidence rate of Thyroid carcinoma (TC) has been increasing worldwide. Thus, research on factors of TC carcinogenesis may promote TC prevention and decrease the incidence rate. There are several studies targeting the correlation between gut microbiota and thyroid disease. Carcinogenesis of several malignancies is influenced by microbiota. However, thyroid microbiome of TC has not been revealed. This study investigated thyroid microbiota in different TC microhabitats.

**Methods:**

We performed 16s rRNA gene sequencing using tumor tissues and matched peritumor tissues from 30 patients with TC to characterize thyroid microbiota.

**Results:**

The richness and diversity of thyroid microbiota were lower in TC tumor samples than in matched peritumor tissues. At the genus level, the core microbiota of thyroid included *Sphingomonas*, *Comamonas*, *Acinetobacter*, *Pseudomonas*, *Microvirgula*, and *Soonwooa*. The abundance of *Sphingomonas* and *Aeromonas* was significantly increased in tumor tissues, while the abundance of *Comamonas*, *Acinetobacter*, and *Peptostreptococcus* was significantly enhanced in peritumor tissues. The combination of *Comamonas* and *Sphingomonas* could discriminate tumor samples from peritumor samples with an area under the curve (AUC) of 0.981 (95% confidence interval [CI] 0.949–1.000). The abundance of *Sphingomonas* was significantly higher in N1 stage than in N0 stage. *Sphingomonas* could distinguish between N0 and N1 stage with an AUC of 0.964 (95% CI 0.907–1.000).

**Conclusions:**

The microbial diversity and composition were significantly different between peritumor and tumor microhabitats from patients with TC, which may eventually affect TC carcinogenesis and progression. The combination of *Comamonas* and *Sphingomonas* could serve as a powerful biomarker for discrimination between tumor and peritumor tissues. Furthermore, the higher abundance of *Sphingomonas* was correlated with lymph node metastasis, indicating that the abundance of *Sphingomonas* may indicate a poor prognosis for TC patients, and *Sphingomonas* may play a role in promoting TC progression.

**Supplementary Information:**

The online version contains supplementary material available at 10.1186/s12967-021-03167-9.

## Introduction

Thyroid carcinoma (TC) is a common endocrine malignancy, with an estimated 567,000 new cancer cases and 41,000 deaths worldwide in 2018 [1]. The main histological types include papillary thyroid carcinoma (PTC), follicular thyroid carcinoma (FTC), Hurthle cell thyroid carcinoma (HCTC), medullary thyroid carcinoma (MTC), and anaplastic thyroid carcinoma (ATC), which account for 80.2%, 11.4%, 3.1%, 3.5%, and 1.7% of thyroid cancer, respectively [2]. In recent years, the incidence rate of TC has been increasing worldwide. Thus, research on factors of TC carcinogenesis may promote TC prevention and decrease the incidence rate.

Several organs, such as lungs, bladder, and urethra, have long been considered sterile. The advent of next-generation sequencing reveals that these organs are inhabited by a robust microbiota [3, 4]. Due to the acidic environment of the human stomach, researchers previously believed that the stomach was not suitable for the growth of other microorganisms and was inhabited exclusively by *Helicobacter pylori*. However, recent advances in sequencing technology make it clear that the stomach is colonized by a huge number of microorganisms [5]. Dysbiosis of gastric microbiota can affect metabolism, inflammation, immunity [6, 7], and eventually result in gastric cancer [8]. The imbalance between the types of microorganisms within the lung can cause lung diseases, such as cystic fibrosis [9], asthma [10], chronic obstructive pulmonary diseases [11], or even lung cancer [12]. However, the profile and functional role of thyroid microbiome in patients with TC has not been revealed.

There are several studies targeting the correlation between gut microbiota and thyroid disease. A study reported that the proportions of *Pasteurellaceae* and *Prevotellaceae* were higher, but the proportions of *Veillonellaceae*, *Enterobacteriaceae*, and *Rikenellaceae* were significantly lower in patients with Graves' disease compared to controls [13]. The abundance of *Lactobacillaceae* and *Bifidobacteria* was reduced, but the abundance of *Enterococcus *spp*.* was increased in hyperthyroid patients compared to healthy controls [14]. Su et al. reported that they observed significant differences in alpha and beta diversities of gut microbiota between patients with primary hypothyroidism and healthy individuals [15]. The fecal microbiota transplantation showed that total thyroxine levels were decreased in mice receiving the transplant from patients with primary hypothyroidism. Gut microbiota diversity and composition were significantly different between patients with TC and healthy controls [16].

The term ‘thyrogastric syndrome’ referring to the link between the gastrointestinal tract and the thyroid has been postulated in 1950s [17]. Gastric mucosal cells and thyroid follicular cells have the same embryonic origin because the thyroid gland develops from primitive gut cells [18]. Thus, we hypothesize that thyroid gland may also be colonized by microorganisms. For the first time, we performed 16s rRNA gene sequencing using tumor tissues and matched peritumor tissues from 30 patients with TC to characterize the core microbiota of thyroid, compare microbial diversity and composition of tumor tissues and matched peritumor tissues, identify differential taxa between tumor tissues and matched peritumor tissues, and characterize the microbial biomarkers for discrimination between tumor and peritumor tissues. The correlation between microbiota of thyroid carcinoma and clinicopathological factors was analyzed.

## Methods

**Fig. 1:**
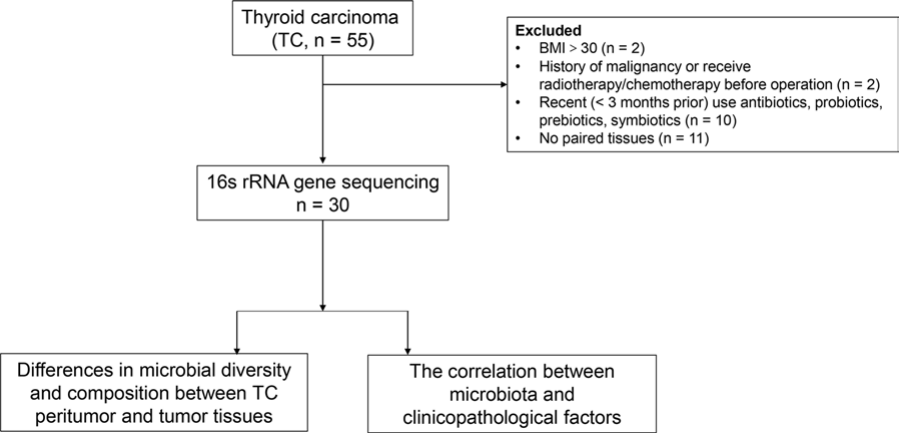
Flowchart explaining enrollment of patients with thyroid carcinoma for this study

This retrospective study included 55 TC patients who underwent total thyroidectomy between March 2018 and December 2018 at the First Affiliated Hospital, School of Medicine, Zhejiang University. Two patients with body mass index (BMI) > 30, two patients with history of malignancy or receiving radiotherapy/chemotherapy before operation, ten patients with recent usage of antibiotics, probiotics, prebiotics, symbiotics, and eleven patients with no paired tissues were excluded (Fig. 1). Finally, 30 subjects were obtained for analysis of thyroid microbiota. The tumor and peritumor (about 3 cm adjacent to the cancer tissue) tissues were collected, which were confirmed by pathological diagnosis. Archival slides of patients were evaluated by two pathologists. We obtained the following clinicopathological information: gender, age upon diagnosis, tumor size, extrathyroidal extension, recurrence risk stratification, and clinical stage. TNM staging was determined based on the 8th edition of the American Joint Committee on Cancer staging system. The clinicopathological information was supplied in Table 1. Approval for this study was obtained from Medical Ethics Committee of the First Affiliated Hospital, School of Medicine, Zhejiang University. Patients signed an informed consent.

**Table 1:** Clinical features of patients with thyroid carcinoma

ID	Histology	Gender	Age	T stage	N stage	M stage	Clinical stage	Extrathyroidal extension	Recurrence risk stratification
P01	PTC	Male	64	T1b	N0	M0	I	Yes	Low risk
P02	PTC	Female	47	T1b	N0	M0	I	No	Low risk
P03	PTC	Female	49	T1a	N0	M0	I	No	Low risk
P04	PTC	Male	34	T1b	N1a	M0	I	No	Intermediate risk
P05	PTC	Female	55	T1a	N0	M0	I	No	Low risk
P06	PTC	Female	46	T1b	N0	M0	I	No	Low risk
P07	PTC	Male	55	T1a	N0	M0	I	No	Low risk
P08	PTC	Male	21	T1b	N1a	M0	I	No	Intermediate risk
P09	PTC	Male	53	T1a	N0	M0	I	No	Low risk
P10	PTC	Male	41	T1b	N1b	M0	I	No	Intermediate risk
P11	PTC	Male	62	T1b	N0	M0	I	No	Low risk
P12	PTC	Female	59	T2	N1	M0	II	No	Low risk
P13	PTC	Female	23	T1b	N0	M0	I	No	Low risk
P14	PTC	Female	60	T1b	N0	M0	I	No	Low risk
P15	PTC	Female	55	T1a	N1a	M0	II	No	Low risk
P16	PTC	Female	54	T1a	N0	M0	I	No	Low risk
P17	PTC	Female	47	T1b	N0	M0	I	No	Low risk
P18	PTC	Female	40	T1b	N1a	M0	I	No	Intermediate risk
P19	PTC	Female	39	T1b	N1a	M0	I	No	Intermediate risk
P20	PTC	Male	29	T1b	N1a	M0	I	No	Intermediate risk
P21	PTC	Female	50	T1b	N0	M0	I	No	Low risk
P22	PTC	Male	34	T1b	N1a	M0	I	No	Intermediate risk
P23	PTC	Female	55	T1b	N1	M0	II	No	Intermediate risk
P24	PTC	Female	52	T1a	N0	M0	I	No	Low risk
P25	PTC	Female	54	T2	N0	M0	I	Yes	Low risk
P26	PTC	Female	25	T1a	N1a	M0	I	No	Intermediate risk
P27	PTC	Female	49	T1a	N0	M0	I	No	Low risk
P28	PTC	Female	47	T1a	N1a	M0	I	No	Intermediate risk
P29	PTC	Female	38	T1b	N1b	M0	I	Yes	Intermediate risk
P30	PTC	Female	33	T1b	N0	M0	I	No	Low risk

PTC: papillary thyroid cancer

### DNA extraction, amplicon library construction, and sequencing

The genomic DNA of thyroid tissues (about 100 mg) was extracted using cetyltrimethylammonium bromide/sodium dodecyl sulfate method. To evaluate environmental contamination, six sterile Petri dishes with sterile water and sterile filter paper were placed in different corners of the operating room for 24 h. The filter paper was transferred to sterile tubes for DNA extraction and subsequent PCR. The extracted DNA from the filter paper was used as quality control (QC). DNA integrity was analyzed by 1% agarose gel electrophoresis. DNA concentration and purity were verified using Nanodrop 2000 (Thermo). We used nested PCR to amplify the V–V4 region of bacterial 16s rRNA gene. During the first round, the 16s rRNA gene was amplified using the 27F (5′-AGAGTTTGATCCTGGCTCAG-3′) and 1492R (5′-GGTTACCTTGTTACGACTT-3′) primers. In the second round, V3-V4 region of 16S rRNA gene was amplified using the 341F (5′-TCGTCGGCAGCGT CAGATGTGTATAAGAGACAGCCTACGGGNGGCWGCAG-3′) and 806R (5′-G TCTCGTGGGCTCGGAGATGTGTATAAGAGACAGGACTACHVGGGTATCTAATCC-3′) primers. All PCR reactions were carried out in 25 μL reaction mixture containing 10 ng of template DNA. Barcode was added using index PCR (Nextera XT Index Kit v2, illumina). The PCR condition of DNA from filter paper was the same as that of DNA from tissues. Index PCR products were sequenced with the Miseq platform.

### Sequencing data analysis

The raw reads were filtered to obtain the high-quality clean reads using USEARCH 8.0. Chimera sequences were detected and removed using the UCHIME algorithm software [19]. Sequences with more than 97% similarity were allocated to one operational taxonomic unit (OTU) using UPARSE software [20]. The phylogenetic affiliation of each 16S rRNA gene sequence was analyzed by RDP Classifier against the Silva 16S rRNA database using confidence threshold of 70%. Subsequent analyses, including alpha diversity analysis, beta diversity analysis on Bray–Curtis distance, the linear discriminant analysis (LDA) effect size (LEfSe), and random forest analysis, were performed using MicrobiomeAnalyst [21].

### Statistical analysis

The statistical Analyses were performed using GraphPad Prism (Version 8.0; GraphPad Software) software. Statistical significance was defined as a two-sided *P*-value of < 0.05. The Mann–Whitney U test was used to calculate the difference in Chao1 index, Shannon index, and the abundance of taxa between two groups.

## Results

### Differences in microbial diversity and composition between TC peritumor and tumor tissues

**Fig. 2:**
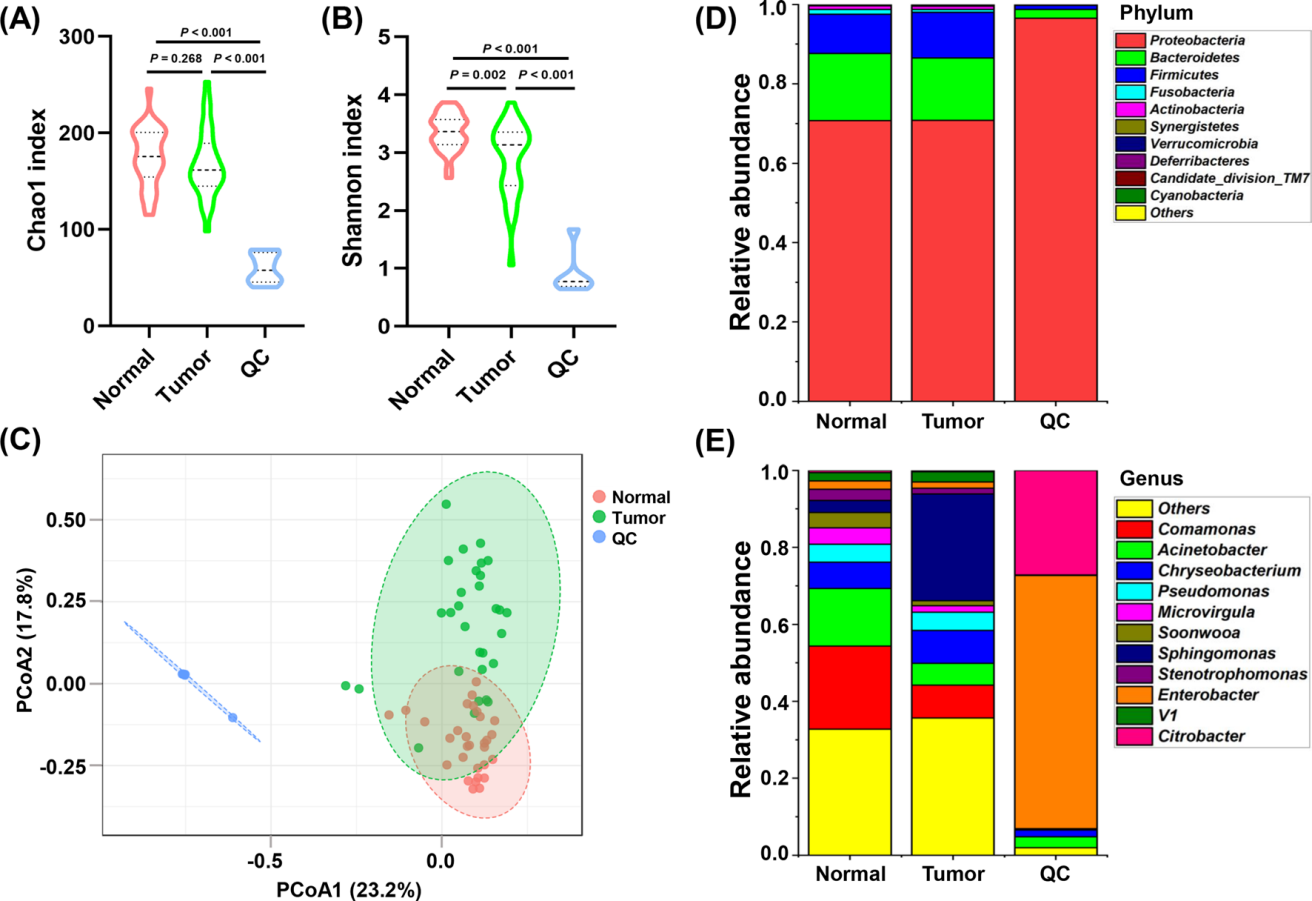
Comparison of microbial profiles between QC samples, TC tumor and matched peritumor tissues before elimination of environmental contamination. **A**, **B** Chao1 and Shannon indices were used to evaluate the microbial diversity of the paired tumor, peritumor tissues, and QC samples. Tumor and matched peritumor tissues were from 30 TC patients. QC samples were used to reveal the environmental microbiota. The Mann–Whitney U test was performed to compare differences between two groups. **C** Principal coordinate analysis (PCoA) of Bray–Curtis analysis demonstrated that QC samples, peritumor and tumor tissues showed three distinct clusters. The microbial relative abundance of TC tumor tissues, matched peritumor tissues, and QC samples at the phylum (**D**) and genus (**E**) levels is shown

As shown in Additional file 1: Table S1, the 16s rRNA gene sequencing produced a median of 41,778 reads for QC samples, 30 paired tumor and peritumor tissues. First, we analyzed alpha diversity to investigate microbial diversity between TC peritumor and tumor tissues. The Chao1 index reflecting species richness was lower in tumor tissues than in peritumor tissues; however, the difference was not significant (*P* = 0.268, Fig. 2A). The Shannon index, which measures species richness and evenness, was significantly lower in tumor tissues in comparison to peritumor tissues (*P* < 0.001, Fig. 2B). To exclude the possibility of contamination from the environment, six QC samples were obtained from the operating room where the tissue samples were collected. The analysis of alpha diversity showed that the Chao1 index and the Shannon index were both significantly lower in QC group compared with peritumor and tumor tissues (*P* < 0.001, Fig. 2A and B).

To compare the composition of the microbial community between peritumor and tumor tissues, beta diversity was analyzed using Bray–Curtis method, and Principal coordinate analysis (PCoA) was performed, which showed that significant clustering was detected between QC and tissues samples. The PCoA also indicated that peritumor and tumor tissues showed two distinct clusters (PERMANOVA, *R*^*2*^ = 0.330, *P* < 0.001, Fig. 2C).

The taxonomic profiles of thyroid microbiota are shown in Fig. 2D and E. We defined the core microbiota of thyroid tissues if it is observed in 80% of samples. At the phylum level, the core microbiota of thyroid was *Proteobacteria*, *Bacteroidetes*, *Firmicutes* (Fig. 2D, Additional file 2: Table S2 and Additional file 3: Table S3). At the genus level, the core microbiota of thyroid included *Comamonas*, *Acinetobacter*, *Chryseobacterium*, *Pseudomonas*, *Microvirgula*, *Soonwooa*, *Sphingomonas* (Fig. 2E, Additional file 4: Table S4 and Additional file 5: Table S5). The proportions of *Comamonas*, *Acinetobacter*, *Microvirgula*, and *Soonwooa* were lower in tumor tissues than in peritumor tissues (Fig. 2E). The tumor tissues had higher abundance of *Sphingomonas* compared with peritumor tissues (Fig. 2E). At the genus level, the core microbiota of QC samples included *Enterobacter*, *Citrobacter*, and *Chryseobacterium*, which were very different from those of thyroid (Fig. 2E).

**Fig. 3:**
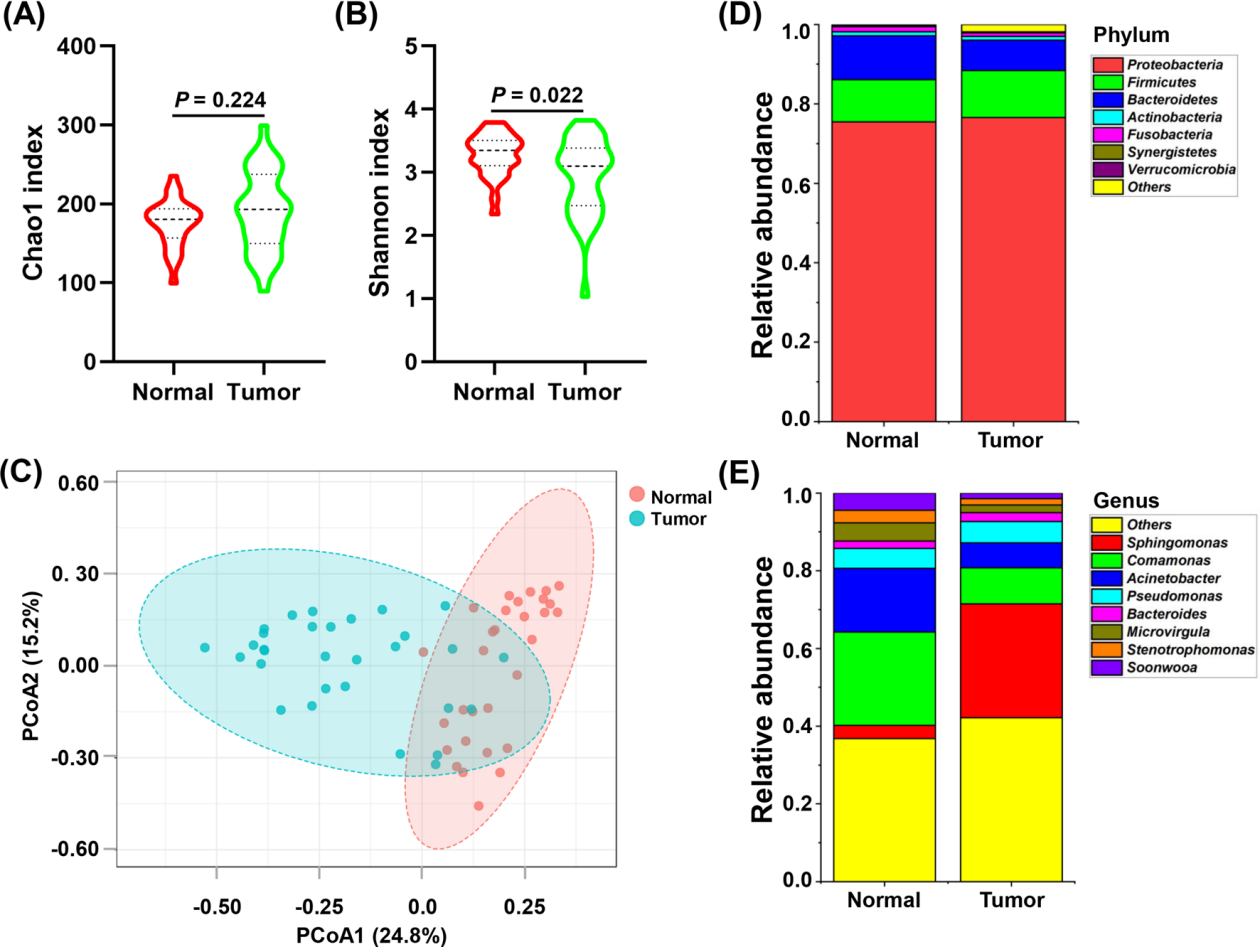
Comparison of microbial profiles between TC tumor and matched peritumor tissues after elimination of environmental contamination. **A**, **B** Comparison of Chao1 and Shannon indices between 30 TC tumor and matched peritumor tissues after elimination of environmental contamination. **C** Principal coordinate analysis (PCoA) demonstrated that the peritumor and tumor tissues showed two distinct clusters. The microbial relative abundance of 30 TC tumor tissues and matched peritumor tissues at the phylum (**D**) and genus (**E**) levels is shown

To exclude the effect of environment on thyroid microbiota, we eliminated OTUs annotated as *Enterobacter*, *Citrobacter*, and *Chryseobacterium* from TC peritumor and tumor tissues. The Chao1 index was higher in tumor tissues than in peritumor tissues; however, the difference was not significant (*P* = 0.224, Fig. 3A). The Shannon index was significantly lower in tumor tissues than in peritumor tissues (*P* = 0.022, Fig. 3B). PCoA showed that significant clustering was detected between tumor and peritumor tissues (PERMANOVA, *R*^*2*^ = 0.162, *P* < 0.001, Fig. 3C). At the phylum level, the core microbiota of thyroid was *Proteobacteria*, *Bacteroidetes*, *Firmicutes* (Fig. 3D). At the genus level, the core microbiota of thyroid included *Sphingomonas*, *Comamonas*, *Acinetobacter*, *Pseudomonas*, *Microvirgula*, and *Soonwooa* (Fig. 3E).

### Determination of differential taxa between different thyroid microhabitats

**Fig. 4:**
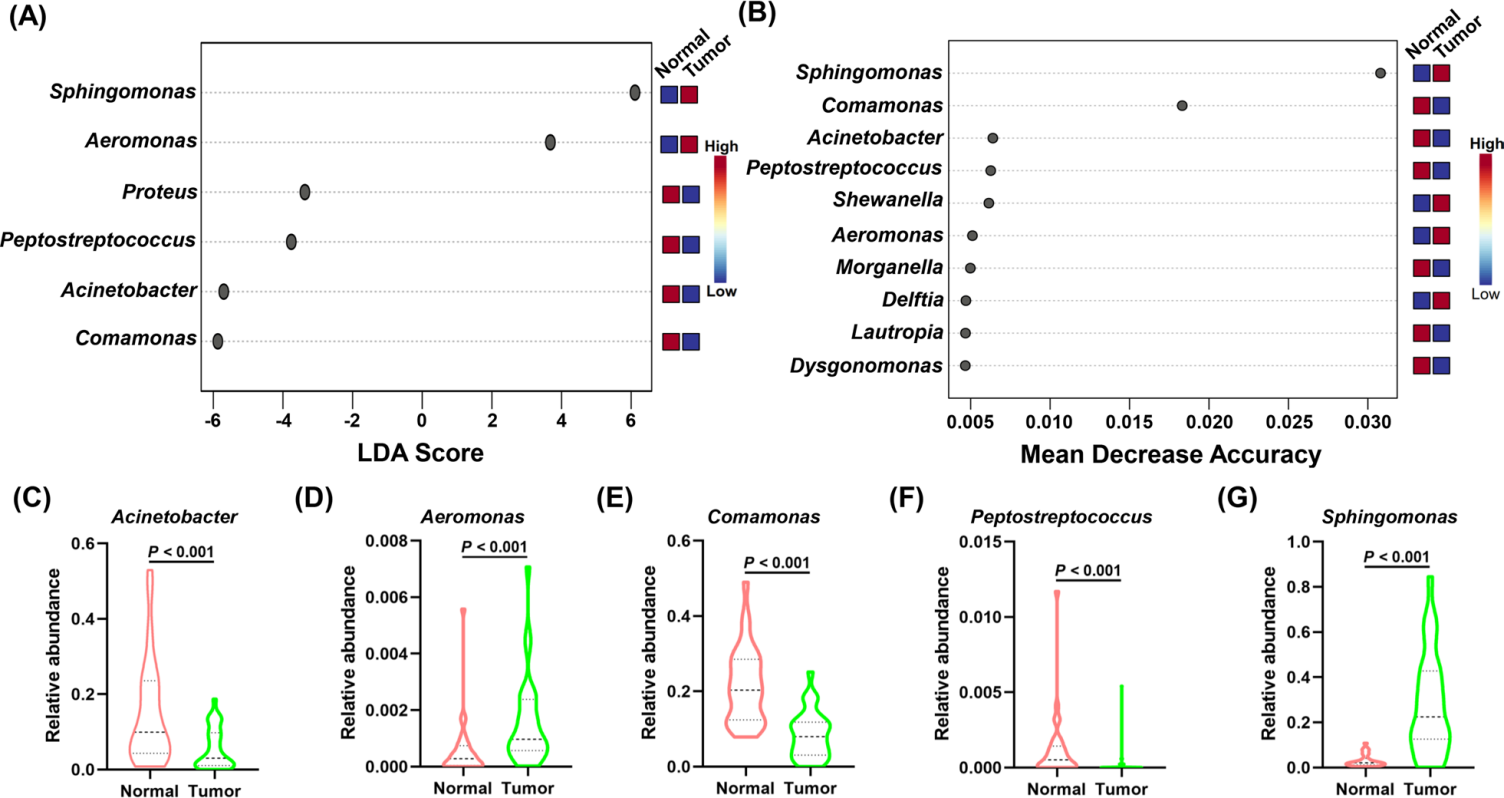
The differential taxa at the genus level between the paired thyroid cancer tissues and peritumor tissues from 30 patients with thyroid cancer. **A** Differential taxa at the genus level identified by linear discriminant analysis (LDA) effect size (LEfSe) analysis (LDA > 3.0, corrected *P* value < 0.05). **B** Differential taxa at the genus level identified by the random forest analysis. **C**–**G** The differential abundance of the 5 discriminative genera between 30 thyroid cancer tissues and matched peritumor tissues was further validated using the Mann–Whitney U test

To identify discriminative taxa between TC peritumor and tumor tissues, we analyzed the compositions of thyroid microbiota in peritumor and tumor tissues using linear discriminant analysis (LDA) effect size (LEfSe) method (LDA > 3.0, corrected *P* value < 0.05). At the phylum level, we did not identify any differential taxa. At the genus level, the abundance of *Sphingomonas* and *Aeromonas* was significantly increased in peritumor tissues, whereas the abundance of *Comamonas*, *Acinetobacter*, *Peptostreptococcus*, and *Proteus* was significantly increased in tumor tissues (Fig. 4A). The 5 differential taxa including *Sphingomonas*, *Aeromonas Comamonas*, *Acinetobacter* and *Peptostreptococcus* were also confirmed by the random forest analysis which revealed 10 differential taxa between peritumor and tumor tissues (Fig. 4B). We further analyzed the differential abundance of the 5 discriminative features at the genus level between the two groups using the Mann–Whitney U test, and demonstrated that the abundance of these taxa was significantly different between the two groups (*P* < 0.05, Fig. 4C–G).

### Identification of different thyroid microhabitats based on thyroid microbiota

**Fig. 5:**
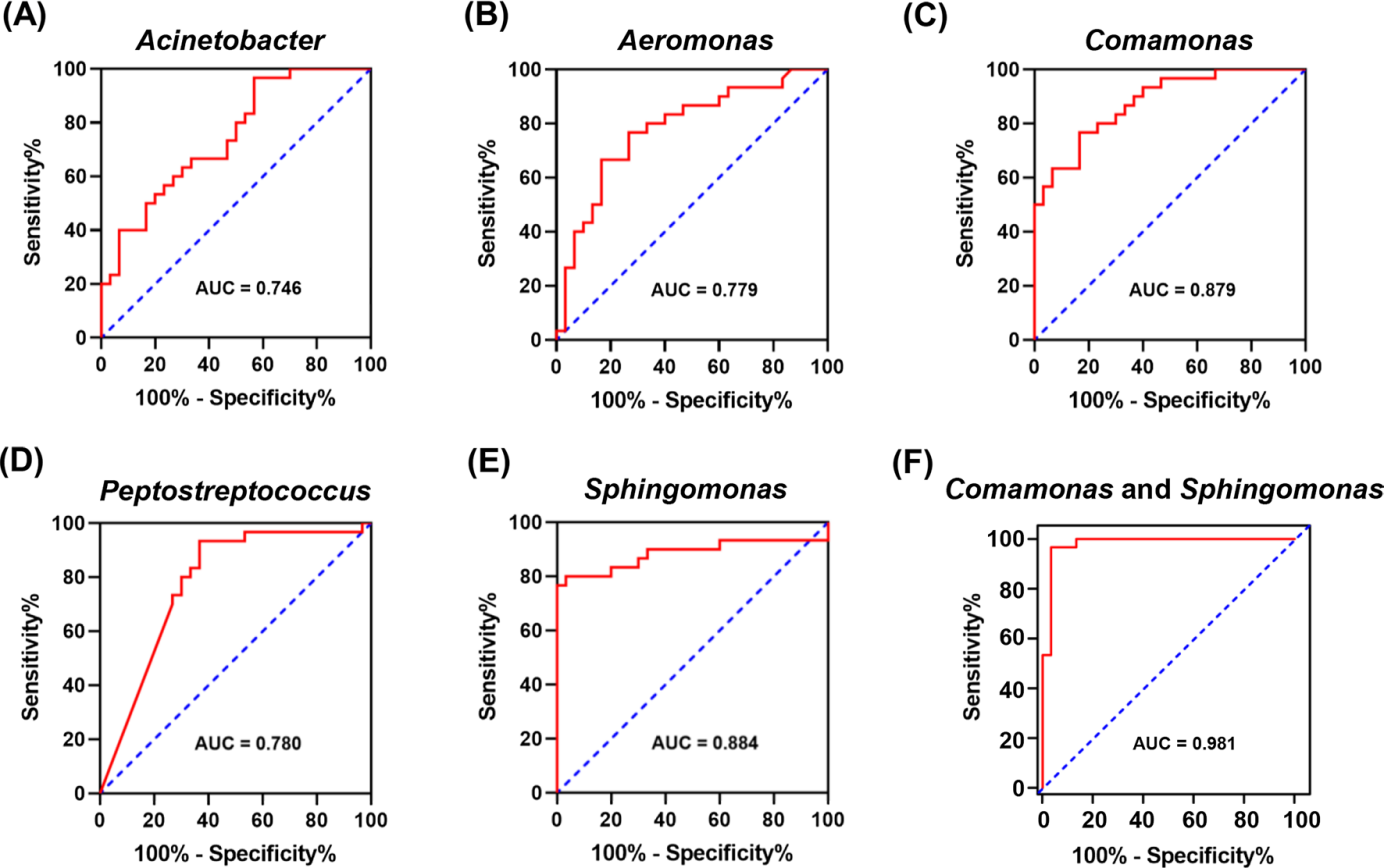
Receiver operating characteristic (ROC) curves for differential genera. **A**–**E** ROC curves for the 5 differential genera and **F** the combination of *Comamonas* and *Sphingomonas* were plotted based on microbial relative abundance

Receiver operating characteristic (ROC) curve analysis was performed to evaluate the diagnostic value of the 5 differential taxa in distinguishing tumor samples from peritumor samples. The areas under the curve (AUCs) of the 5 taxa ranged from 0.746 to 0.884 (Fig. 5). *Comamonas* and *Sphingomonas* had an AUC value > 0.800 (Fig. 5C and 5E), and were further selected as the potential biomarkers. The combination of *Comamonas* and *Sphingomonas* could rapidly increase the diagnostic accuracy in discriminating tumor samples from peritumor samples with an AUC value of 0.981 (95% confidence interval [CI] 0.949–1.000, Fig. 5F).

### The association between thyroid microbiota and clinicopathological factors

**Fig. 6:**
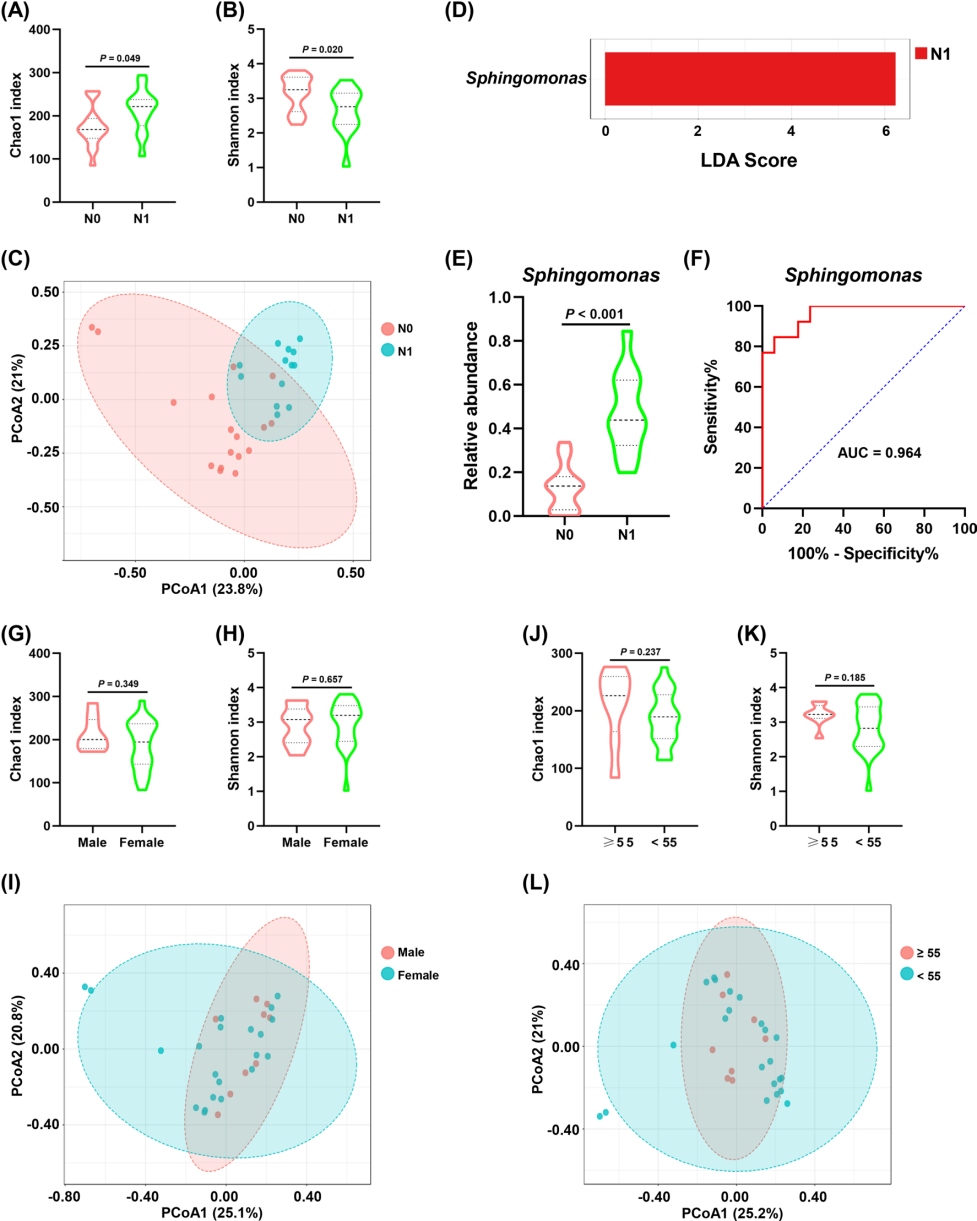
The association between clinicopathological factors and thyroid microbiota. **A**, **B** The differences in Chao1 and Shannon indices between tumor tissues from thyroid cancer patients at N0 and N1 stage. Mann–Whitney U tests were performed. **C** Principal coordinate analysis (PCoA) based on Bray–Curtis distance revealed that thyroid cancer patients at N0 stage were significantly different from those at N1 stage. **D** Linear discriminant analysis (LDA) effect size (LEfSe) analysis (LDA > 3.0, corrected *P* value < 0.05) was performed to evaluate differential taxa at the genus level. **E** The differential abundance of *Sphingomonas* between N0 and N1 stage was further validated using the Mann–Whitney U test. **F** The receiver operating characteristic (ROC) curve for *Sphingomonas* genera was plotted using microbial relative abundance to assess the value of thyroid microbiota as a diagnostic tool to distinguish between thyroid patients with N0 and N1 stage. **G**, **H** The differences in Chao1 and Shannon indices between tumor tissues from male and female patients with thyroid cancer. Mann–Whitney U tests were performed. **I** Principal coordinate analysis (PCoA) of thyroid microbiota in male and female patients with thyroid cancer based on Bray–Curtis distance. **J**, **K** The differences in Chao1 and Shannon indices between tumor tissues from thyroid cancer patients aged ≥ 55 and < 55. Mann–Whitney U tests were performed. **L** Principal coordinate analysis (PCoA) of thyroid microbiota in patients with thyroid cancer aged ≥ 55 and < 55 based on Bray–Curtis distance

To reveal the association between lymph node metastasis and thyroid microbiome, we analyzed microbial differences between tumor tissues from patients at N0 and N1 stage. The Chao1 index was significantly higher in N1 stage than in N0 stage (*P* = 0.049, Fig. 6A), while the Shannon index was significantly lower in N1 stage in comparison to N0 stage (*P* = 0.020, Fig. 6B). PCoA analysis based on the Bray–Curtis method showed that significant clustering was detected between patients at N0 and N1 stage (PERMANOVA, R^2^ = 0.164, *P* < 0.001, Fig. 6C). The LEfSe analysis showed that the abundance of *Sphingomonas* was significantly increased in N1 stage compared to N0 stage (Fig. 6D), which was also confirmed by the Mann–Whitney U test (*P* < 0.001, Fig. 6E). ROC curve analysis showed that *Sphingomonas* could distinguish between patients at N0 and N1 stage with an AUC of 0.964 (95% CI 0.907–1.000, Fig. 6F). However, we observed no significant differences in Chao1 and Shannon indices between male and female patients (*P* = 0.349, Fig. 6G; *P* = 0.657, Fig. 6H). PCoA analysis suggested that there was no significant difference in composition of thyroid microbiota between male and female patients (PERMANOVA, R^2^ = 0.033, *P* = 0.473, Fig. 6I). LEfSe analysis revealed no differential taxa between male and female patients. Figure 6J–L showed that there was no difference in the diversity and composition of thyroid microbiota between patients aged ≥ 55 and < 55.

## Discussion

In this study, we used 16s rRNA gene sequencing to characterize thyroid microbiota in different thyroid microhabitats. The alpha diversity and beta diversity were both significantly different between QC samples and thyroid tissue samples, indicating that thyroid tissues were not contaminated by the surrounding environment. We found that TC tumor tissues had lower thyroid microbiota richness and diversity than matched peritumor tissues. A decrease in microbiota diversity in tumor tissues was also observed in patients with lung cancer and gastric cancer [22, 23]. Nevertheless, an increase in gut microbiota diversity is observed in patients with TC, Hashimoto's thyroiditis, and hyperthyroidism [14, 16, 24]. At the genus level, the core microbiota of thyroid tissues included *Comamonas*, *Acinetobacter*, *Pseudomonas*, *Microvirgula*, *Soonwooa*, and *Sphingomonas*, while the core gut microbiota of TC encompassed *Faecalibacterium*, *Bacteroides*, *Blautia*, *Rosebulia*, *Dialister*, and *Lachnoclostridium*. These results showed that the composition of thyroid microbiota and gut microbiota of TC patients were different.

We identified 5 differential taxa, including the genus *Sphingomonas* and *Aeromonas* enriched in peritumor tissues, and *Comamonas*, *Acinetobacter* and *Peptostreptococcus* enriched in tumor tissues. However, the comparison of the gut microbial compositions between TC and healthy subjects showed that 27 genera, including *Bacteroides*, *Roseburia*, *Megamonas*, *Klebsiella*, *Blautia*, etc., markedly differed between the two groups with significantly differential abundance. These results demonstrated that the differential taxa of thyroid microbiota and gut microbiota were quite different, indicating different roles of thyroid microbiota and gut microbiota in promoting TC development.

More importantly, the higher abundance of *Sphingomonas* was associated with lymph node metastasis. In addition, patients enrolled in this study were at early stage (stage I and II). These results indicated that the abundance of *Sphingomonas* may represent a prognostic value for TC patients at early stage, and *Sphingomonas* may play a role in promoting TC development. An analysis of the global mucosa-associated microbiota revealed that the abundance of *Sphingomonas* was found to be increased in patients suffering colitis associated cancer compared with those with sporadic cancer [25]. Jeong et al. reported that the higher level of *Comamonas* in tumor tissues was associated with more metastasized lymph nodes in pancreatic cancer [26]. *Comamonas* is a cellulolytic microbe that could impact the host metabolism in cancer patients and play a role in inflammation [27]. Ling et al. reported that *Comamonas* was negatively associated with BDCA2 + pDCs, indicating its correlation with antitumor immunity [28]. These studies showed that *Comamonas* and *Sphingomonas* may play important roles in tumor progression. Furthermore, we found that the combination of *Comamonas* and *Sphingomonas* could discriminate tumor samples from peritumor samples with an AUC value of 0.981, indicating that the combination may be a powerful biomarker for TC.

A metagenomic analysis of the stool samples showed that the abundance of *Acinetobacter* was decreased in patients with colorectal cancer compared with healthy subjects [29]. A 16s rRNA gene sequencing analysis of lung tissues revealed that the microbial community compositions of patients only with emphysema were characterized by a significantly higher abundance of *Proteobacteria* (primary the genus *Acinetobacter*) in comparison to lung cancer patients with or without emphysema [23]. *Acinetobacter* is widespread in natural environments and plays an important role in disseminating infections, including the respiratory tract and urinary tract. Certain species of *Acinetobacter* are resistant to multi-drugs and regarded as important pathogens. *Acinetobacter baumannii* is an important opportunistic pathogen that is ubiquitous in hospitals and other settings related with healthcare. A systematic review of thirteen original articles showed that gastric carcinogenesis could be associated with an increase in the abundance of *Acinetobacter baumannii* [30]. These studies showed that the genus *Acinetobacter* could be related with carcinogenesis of different malignancies. We found that the genus *Acinetobacter* was enriched in TC tumor tissues, indicating that it may promote TC progression.

Meanwhile, this study has a few limitations. First, the sample size in this study was small. This study has obtained several positive results, which pave the way for future study with larger sample size. The quantitative reverse-transcription polymerase chain reaction (RT-qPCR) assay is a common quantitative approach to identify microbes in tumor tissues. In the future, we will utilize RT-qPCR to quantitatively identify bacteria, particularly *Comamonas* and *Sphingomonas*, in thyroid cancer tissues in larger sample size. Second, this was a retrospective study, and a prospective study is needed to validate our results. Third, the lack of a control group with benign nodules may compromise the interpretation of the results, since comparing the microbial difference of thyroid tissues between patients with benign nodules and TC will further support the observations of this work. In addition, 16s rRNA sequencing cannot identify the specific bacterial species, resulting in that information on the species of thyroid microbiota was not obtained. Thus, a study revealing the species of thyroid microbiota is needed in future.

## Conclusion

Taken together, we found that the richness and diversity of thyroid microbiota were significantly lower in TC tumor samples in comparison with matched peritumor tissues. The abundance of genus *Sphingomonas* and *Aeromonas* was increased in tumor tissues, while the abundance of *Comamonas*, *Acinetobacter*, and *Peptostreptococcus* was enhanced in peritumor tissues. The combination of *Sphingomonas* and *Comamonas* could serve as a powerful marker for distinguishing TC tumor tissues from matched peritumor tissues. Furthermore, the increased abundance of *Sphingomonas* was correlated with lymph node metastasis, indicating that the abundance of *Sphingomonas* may represent a prognostic value for TC patients at early stage, and *Sphingomonas* may play a role in promoting TC development.

## Supplementary Information

**Additional file 1: Table S1.** Sequencing reads of samples by 16s rRNA sequencing.

**Additional file 2: Table S2.** The identified phylum in each samples.

**Additional file 3: Table S3.** The identified phylum in each group.

**Additional file 4: Table S4.** The identified genera in each sample.

**Additional file 5: Table S5.** The identified genera in each group.

## Data Availability

The original data presented in the study are included in the article, further inquiries can be directed to the corresponding authors.

## Abbreviations

ATC: Anaplastic thyroid carcinoma
AUC: Area under the curve
BMI: Body mass index
CI: Confidence interval
FTC: Follicular thyroid carcinoma
HCTC: Hurthle cell thyroid carcinoma
LEfSe: Linear discriminant analysis (LDA) effect size
MTC: Medullary thyroid carcinoma
OTU: Operational taxonomic unit
PCoA: Principal coordinate analysis
PTC: Papillary thyroid carcinoma
QC: Quality control
ROC: Receiver operating characteristic
TC: Thyroid carcinoma
