# Supplementary material for: Alterations of thyroid microbiota across different thyroid microhabitats in patients with thyroid carcinoma
Source: J Transl Med. 2021 Nov 30;19:488. doi: 10.1186/s12967-021-03167-9 (PMC8638380; doi:10.1186/s12967-021-03167-9)
Supplement: Supplementary file 1 — Additional file 1: Table S1. Sequencing reads of samples by 16s rRNA sequencing. [file 12967_2021_3167_MOESM1_ESM.docx]

Table S1. Sequencing reads of samples by 16s rRNA sequencing.

| **Sample_ID** | **Reads** | **Total_base** | **Average** |
| --- | --- | --- | --- |
| Normal_1 | 38425 | 17868892 | 465 |
| Normal_10 | 37574 | 17437135 | 464.1 |
| Normal_11 | 56149 | 26041231 | 463.8 |
| Normal_12 | 26746 | 12408664 | 463.9 |
| Normal_13 | 31480 | 14585562 | 463.3 |
| Normal_14 | 66887 | 31241330 | 467.1 |
| Normal_15 | 66202 | 30867344 | 466.3 |
| Normal_16 | 31002 | 14342220 | 462.6 |
| Normal_17 | 28599 | 13194048 | 461.3 |
| Normal_18 | 41768 | 19442767 | 465.5 |
| Normal_19 | 48394 | 22557985 | 466.1 |
| Normal_2 | 64629 | 29841856 | 461.7 |
| Normal_20 | 42572 | 19688713 | 462.5 |
| Normal_21 | 37290 | 17155742 | 460.1 |
| Normal_22 | 76164 | 35421494 | 465.1 |
| Normal_23 | 23465 | 10916520 | 465.2 |
| Normal_24 | 27256 | 12662667 | 464.6 |
| Normal_25 | 35328 | 16405425 | 464.4 |
| Normal_26 | 27705 | 12854573 | 464 |
| Normal_27 | 22295 | 10347034 | 464.1 |
| Normal_28 | 33114 | 15438501 | 466.2 |
| Normal_29 | 43025 | 20033391 | 465.6 |
| Normal_3 | 34456 | 15820690 | 459.2 |
| Normal_30 | 44706 | 20902660 | 467.6 |
| Normal_4 | 28358 | 13054020 | 460.3 |
| Normal_5 | 88530 | 40772384 | 460.5 |
| Normal_6 | 67505 | 31320924 | 464 |
| Normal_7 | 40739 | 18900572 | 463.9 |
| Normal_8 | 31035 | 14374753 | 463.2 |
| Normal_9 | 39475 | 18361230 | 465.1 |
| QC_1 | 46200 | 21661699 | 468.9 |
| QC_2 | 42653 | 19990490 | 468.7 |
| QC_3 | 44827 | 21015063 | 468.8 |
| QC_4 | 54610 | 25590726 | 468.6 |
| QC_5 | 44425 | 20819036 | 468.6 |
| QC_6 | 55746 | 26104644 | 468.3 |
| Tumor_1 | 16570 | 7483098 | 451.6 |
| Tumor_10 | 120586 | 55843088 | 463.1 |
| Tumor_11 | 47328 | 21702644 | 458.6 |
| Tumor_12 | 31498 | 14357981 | 455.8 |
| Tumor_13 | 62266 | 28664169 | 460.4 |
| Tumor_14 | 37399 | 17008993 | 454.8 |
| Tumor_15 | 69169 | 31701703 | 458.3 |
| Tumor_16 | 49149 | 22645269 | 460.7 |
| Tumor_17 | 26661 | 12170084 | 456.5 |
| Tumor_18 | 42042 | 18987171 | 451.6 |
| Tumor_19 | 72277 | 33226708 | 459.7 |
| Tumor_2 | 32714 | 15000113 | 458.5 |
| Tumor_20 | 69322 | 31642792 | 456.5 |
| Tumor_21 | 45147 | 20536010 | 454.9 |
| Tumor_22 | 30687 | 13716670 | 447 |
| Tumor_23 | 50341 | 23106865 | 459 |
| Tumor_24 | 24863 | 11548010 | 464.5 |
| Tumor_25 | 45879 | 21294132 | 464.1 |
| Tumor_26 | 58173 | 26727688 | 459.5 |
| Tumor_27 | 35533 | 16046493 | 451.6 |
| Tumor_28 | 40353 | 18216754 | 451.4 |
| Tumor_29 | 66681 | 30914218 | 463.6 |
| Tumor_3 | 36289 | 16435034 | 452.9 |
| Tumor_30 | 86047 | 39911094 | 463.8 |
| Tumor_4 | 52545 | 23800861 | 453 |
| Tumor_5 | 41091 | 18718440 | 455.5 |
| Tumor_6 | 33196 | 14966116 | 450.8 |
| Tumor_7 | 41787 | 19250473 | 460.7 |
| Tumor_8 | 18342 | 8556218 | 466.5 |
| Tumor_9 | 21237 | 9648829 | 454.3 |
